# Guidance for Ophthalmologists and Ophthalmology Centers during the COVID-19 Pandemic

**DOI:** 10.18502/jovr.v15i3.7466

**Published:** 2020-07-29

**Authors:** Zhale Rajavi, Sare Safi, Maryam Mohammadzadeh

**Affiliations:** ^1^Ophthalmic Epidemiology Research Center, Research Institute for Ophthalmology and Vision Science, Shahid Beheshti University of Medical Sciences, Tehran, Iran; ^2^Ophthalmic Research Center, Research Institute for Ophthalmology and Vision Science, Shahid Beheshti University of Medical Sciences, Tehran, Iran

Dear Editor,

Coronavirus Disease (COVID-19) caused by the SARS-CoV-2 virus usually manifests with respiratory symptoms including cough and dyspnea associated with fever and diarrhea, while it rarely presents with conjunctivitis. Ophthalmologists are at high risk for being infected or transmitting the virus due to the following reasons: examining patients with conjunctivitis or asymptomatic carriers from a short distance (usually < 20 cm), the long duration of eye examinations/procedures (> 15 min), touching patients' eyelids during slit-lamp examination, and the possibility of viral shedding and transmission in contact with ocular secretions. During the pandemic peak of COVID-19 in March 2020, a guidance was prepared by the Knowledge Management Unit at the Ophthalmic Research Center, Research Institute for Ophthalmology and Vision Science, Shahid Beheshti University of Medical Sciences, Tehran, Iran for ophthalmologists and eye care centers.^[1]^ We also discuss on necessary precautions for the post-peak period starting from May 2020 and during the partial recovery phase of the COVID-19 pandemic.

##  Characteristics 

•Person to person transmission is caused by respiratory droplets produced by cough and sneezes of the patient. Furthermore, the virus can spread when someone touches infected surfaces and then touches their nose, mouth, and eyes.^[1,2]^

•Symptoms can manifest early (in 2 days) or late (within 14 days) after exposure to the virus. Since the incubation period is between 5 and 7 days, the quarantine period is considered to be at least 14 days.

•Even a carrier without any respiratory symptoms can transmit the infection.

•The virus could survive for 24 hours on cardboard surfaces and credit cards, while it lasts for two–three days on plastic and metal surfaces.

•No vaccines have been produced against the virus so far and research projects in this field are in process.

•Currently, there is no specific drug for prophylaxis and treatment of this virus.

##  Risk factors

The risk of COVID-19 infection is higher in persons with the following characteristics:

•Age > 55 years

•History of respiratory disease

•History of renal disease

•Diabetes (HbA1C > 7.6%)

•History of hypertension

•History of cardiopulmonary disease

•History of organ transplant and use of immunosuppressive drugs

##  General precautions

It is very important to follow routine health instructions against this virus such as washing hands frequently, disinfecting surfaces, and using personal protection equipment (PPE).^[1,2][3][4][5,6]^

##  Recommendations for ophthalmologists in the epidemic peak of March 2020

•Cancel previously scheduled appointments and avoid scheduling new appointments for future months.

•Provide facilities for telemedicine communications with patients for further consultations, answering their questions, and dose adjustment of their medications.^[3,5,6]^

•In case of unavoidable in-person appointments, ask the patient during a phone contact about symptoms such as cough, sneezing, fever, dyspnea, and traveling to high-risk regions. If their answer is yes, avoid visiting the patient at least for three weeks.^[1,2][3][4][5,6]^

•The patients should be examined alone (without companions) and if needed only one person can accompany elderly patients and children.^[6]^

•Avoid crowding in the waiting room while maintaining at least 3 m distance between patients. If possible, encourage patients to wait for the appointment outside the facilities (i.e., in their cars) and notify them when they should return to the office.^[3,6]^

•It is recommended to screen patients and their companions for fever and respiratory symptoms and ask them to wear masks and wash their hands before entering the examination room. Suspicious cases should be referred to relevant healthcare centers.^[6]^

•Limit ophthalmic examinations to urgent cases such as retinal detachment, trauma, chemical contact, and severe ocular infections. It is recommended to perform the examination faster and also avoid unnecessary ones.^[5,6]^

•It is recommended to postpone non-urgent examinations and elective surgeries.

•It is necessary for the ophthalmologists to wear masks during the examination and it is also essential for doctors and health caregivers to wash their hands after contact with every patient.^[1,2][3][4][5,6]^

•In order to prevent exposure to patients' respiratory droplets, it is recommended to use slit-lamp shield.^[1,5]^

•Avoid talking with patients during the examination.

•To prevent the patient to patient transmission, single-dose drops are preferred.^[6]^

•Use applicators to examine the eyelids.

•If possible, use non-contact tonometer to measure the intraocular pressure.^[3,6]^ If not possible, disinfect the tip of Goldmann applanation as follows. First of all, wash the tonometer with water and soap, then allow it to dry. Afterward, put almost 2 mm of its tip in the bleach-based solution with 5% concentration. Isopropyl alcohol and ethyl alcohol are also frequently used; however, they are not as effective as the aforementioned method.

•It is recommended to disinfect the examination room, all equipment including slit-lamp and its accessories after every visit.^[3,6]^

•Use disposable gloves when sanitizing surfaces and equipment.

•Use bleach-based disinfectants (with a concentration of one spoon in a gallon of water) for disinfecting surfaces and use alcohol 70% for instruments.

•If possible, use telemedicine communication such as video or telephone consultations.^[3,5]^

•When examining confirmed or suspected cases, ophthalmologists should wear N95 face masks, goggles or face-protective shield, gloves, gown, and disposable overshoes.

•If any of the hospitalized patients need urgent diagnostic or therapeutic measures, in order to avoid patient transport, it is recommended to perform necessary procedures at the host hospital. If further examinations or urgent surgery is needed, refer the patient to an equipped ophthalmic center.

##  Recommendations for Ophthalmic Centres in the epidemic peak in March 2020

•It is recommended that managers assure their healthcare workers that all clients are going to be screened on arrival.^[5]^

•Managers should monitor cancelation of routine examinations and elective procedures. Also, they should prevent companions from entering the center and keep the waiting rooms uncrowded. If necessary, each patient may only have one companion who should be screened in a similar manner as patients.^[3,6]^

•Provide facilities for telemedicine consultations through phone calls, video conferences, and social networks.^[3,5]^

•Healthcare personnel working hours should be scheduled to minimize work fatigue.^[5]^

•Educate personnel to distinguish and isolate high-risk patients.

•Educate and monitor the process of disinfection of ophthalmic instruments, surfaces, chairs, and elevator handles regularly.^[3,6]^

•Give leave to non-essential personnel in each working shift.

•Provide enough disinfectants including soap, tissue paper, masks, disposable gloves, 70% alcohol, disinfectant gel, and thermometers for patients and healthcare workers.^[1,2][3][4][5,6]^

•Supply slit-lamp shields, goggles, and counter shields.^[3,5]^

•Administrate social distancing while in self-service dining rooms and handover foods in packages.^[3,6]^

##  Recommendations for ophthalmologists and ophthalmic centres during the post-peak period 

All practitioners should follow these recommendations until Food and Drug Administration (FDA)-approved treatments and/or vaccines for COVID-19 are available:

• Respect social distancing.

• Both patients and healthcare workers should utilize face masks.

• Maintain a lower in-office appointment numbers than that in the pre-COVID-19 period.

•Lengthen the turnover time in the operating room.

• Consider cataract surgery as a semi-urgent surgery, not elective, when the patient cannot drive or work or has a risk of falling.

•It is recommended to test ophthalmologists, health caregivers, and patients prior to elective visits and surgeries. The tests and their characteristics are:

RT-PCR which is usually done on nasopharyngeal swab specimens can be positive even after 35 days since the onset of symptoms.

The specificity of the SARS-CoV-2 antibody test is 99.8% and has no cross-reactivity to other coronaviruses. A positive serology test shows recent infection. However, the virus can shed for at least five weeks from the onset of the infection. The duration and degree of protection of the IgG antibody response from reinfection are unknown. In regions where the prevalence of COVID-19 is low, positive serologic tests, without prior COVID-19 (RT-PCR) positive test, is more probable to be an artifact or the testing error rather than true infections.

A rapid antigen detection test is available but is more probable to report false-negative results and needs a fluorescent immunoassay analyzer to be acceptable.

•In the case of in-office procedures that necessitate close interaction between the surgeon and patients, wearing surgical masks for both patients and surgeons is recommended. Surgeons are also encouraged to wear eye protection and N95 masks.

•During procedures needing general anesthesia (GA), caregivers without N95 mask should remain out of the operating room throughout intubation/extubation. Furthermore, for procedures with monitored anesthesia/conscious sedation, the patient should wear a surgical mask. Due to prolonged physical proximity between the ophthalmologist and the patient during surgery, it is recommended for the surgeon to wear an N95 mask.

As time goes by, ophthalmologists will be required to perform routine in-office procedures and examine patients who have recovered or are recovering from COVID-19. Due to prolonged viral shedding (even reported for up to 37 days), it is recommended to repeat RT-PCR testing for patients who are being scheduled for operation < 6 weeks after their diagnosis (except for emergency cases).

If the repeat test is positive, the patient should wear a surgical mask and the surgeon should wear an N-95 mask, gown, and eye protection.

##  Financial Support and Sponsorship

Nil.

##  Conflicts of Interest

There are no conflicts of interest.
